# Comparison of quality/quantity mNGS and usual mNGS for pathogen detection in suspected pulmonary infections

**DOI:** 10.3389/fcimb.2023.1184245

**Published:** 2023-07-31

**Authors:** Zhan Zhao, Xuefen Chen, Yubao Wang, Jing Feng

**Affiliations:** ^1^ Respiratory Department, Tianjin Medical University General Hospital, Tianjin, China; ^2^ Department of Respiratory Medicine, Characteristic Medical Center of the Chinese People’s Armed Police Force, Tianjin, China

**Keywords:** usual metagenomic next-generation sequencing, quality/quantity metagenomic next-generation sequencing, pulmonary infection, pathogen detection, pulmonary pathogens

## Abstract

Improved metagenomic next-generation sequencing (mNGS), for example, quality/quantity mNGS (QmNGS), is being used in the diagnosis of pulmonary pathogens. There are differences between QmNGS and the usual mNGS (UmNGS), but reports that compare their detection performances are rare. In this prospective study of patients enrolled between December 2021 and March 2022, the bronchoalveolar lavage fluid of thirty-six patients with suspected pulmonary infection was assessed using UmNGS and QmNGS. The sensitivity of QmNGS was similar to that of UmNGS. The specificity of QmNGS was higher than that of UmNGS; however, the difference was not statistically significant. The positive likelihood ratios (+LR) of QmNGS and UmNGS were 3.956 and 1.394, respectively, and the negative likelihood ratios (-LR) were 0.342 and 0.527, respectively. For the co-detection of pathogens, the depth and coverage of the QmNGS sequencing were lower than those of UmNGS, while for the detection of pathogens isolated from patients with pulmonary infection, the concordance rate was 77.2%. In the eleven patients with nonpulmonary infection, only viruses were detected using QmNGS, while UmNGS detected not only viruses but also bacteria and fungi. This study provides a basis for the selection of mNGS for the diagnosis of suspected pulmonary infection.

## 1 Introduction

A pulmonary infection is a common respiratory disease that remains prevalent worldwide, with substantial mortality and morbidity (Wunderink and Waterer, 2017). It is estimated that approximately 5.6 million people acquire pulmonary infections each year in USA. The annual cost associated with pulmonary infections is approximately over $12 billion (Colice et al., 2004). This not only brings a burden on the economy, but also puts a tremendous stress on public health. Numerous pathogens can cause pneumonia, making diagnosis challenging. Furthermore, early and effective antibacterial therapy is crucial for the prognosis of immunocompromised individuals and older individuals susceptible to pneumonia. Therefore, the clinical requirements for a rapid and accurate etiological diagnosis to improve patient outcomes are increasing. Typically, cultures, which are the gold standard for microbial identification, have long turnaround times, low sensitivity, and are limited to certain pathogens. Culture-independent pathogen detection methods such as immunological assays and nucleic acid amplification tests target only a fraction of the currently known pathogens (Murdoch et al., 2012). This often leads to delay clinical treatment. Metagenomic next-generation sequencing (mNGS) as a promising culture-independent technique demonstrates advantages such as rapidity for the pathogenic diagnosis of pulmonary infections.

mNGS, which combines high-throughput sequencing and bioinformatic analysis to detect microbial species and their abundances based on the BLAST database, is an unbiased detection method with high efficiency and a short turnaround time. mNGS shows good performance for pathogen diagnosis in both single and mixed pulmonary infections (Zhao et al., 2021). Although this detection technology has obvious advantages, it also faces significant challenges regarding sensitivity, interpretation, turnaround time, and laboratory workflow. In a pulmonary infection, since the content of human-derived DNA varies greatly in different samples and the diagnostic sensitivity of mNGS is affected by the content of human-derived DNA (Schlaberg et al., 2017), the ability to distinguish true pathogens from respiratory colonization and environmental contamination is hindered (Chen et al., 2020). Thus, it is important to know how to balance the human-derived DNA depletion techniques that are currently used to increase the sensitivity of mNGS, as using these techniques can lead to nonspecific clearance of pathogens (Charalampous et al., 2019). The process of an mNGS experiment is complex, and it is relatively difficult to verify the entire workflow and establish a stable quality control system. In addition, different sequencing platforms and bioinformatics pipelines applied by mNGS may affect the accuracy and reproducibility of the detection results. Currently, a few studies have attempted to compensate for the shortcomings of the current mNGS technology, among which quality/quantity mNGS (QmNGS) is the most representative. QmNGS is based on using selective human-derived DNA, a polymerase chain reaction (PCR)-free library preparation, and the spiking of constant concentrations of a nucleic acid internal control (IC) into all specimens to detect microbial abundance and cellularity. A wet laboratory workflow of QmNGS has been performed using an automated device (NGSmaster) (Luan et al., 2021). Although QmNGS claims have been said to reduce false negatives, its efficacy in the diagnosis of clinical pulmonary infection pathogens is unknown.

In this study, our aim was to compare the differences in etiological diagnoses such as diagnostic accuracy, concordance rate and sequencing depth and coverage for the co-detection of pathogens between QmNGS and usual mNGS (UmNGS). UmNGS is currently the most common mNGS technology. It is based on indiscriminate preparation of human-derived DNA and PCR-based library preparation. By comparing the effects of the different mNGS detection methods on the detection of clinical pulmonary infection pathogens, we further aimed to provide a more theoretical basis for clinical selection.

## 2 Materials and methods

### 2.1 Patients and specimen collection

Our prospective study included patients with a high suspicion of pulmonary infections who were admitted to the Tianjin Medical University General Hospital between November 2021 and March 2022. Patients were excluded based on poor coagulation, the presence of cardiac disease not allowing bronchoscopy, or refusal to participate in the study, and thirty-six patients were ultimately enrolled in this study. CT scans, sputum smear microscopy and culture, galactomannan test, (1,3)-β-D-glucan test, and other etiological tests according to their condition were performed on all patients. Furthermore, all patients underwent bronchoscopy and BALF was collected from lung lesions. A portion of the BALF was used for pathogen identification using cultures, smears, and Xpert. The remaining BALF was divided equally into two aliquots, each containing 4 ml, and stored at 4°C for mNGS detection. The demographic characteristics and related underlying diseases of the patients were collected from their medical records. Three or more professional physicians comprehensively determined the final clinical diagnosis of the patient according to the patient’s clinical symptoms, signs, imaging, microbiology, and etiology results. The study was in accordance with the Ethics Review Board requirements of the Tianjin Medical University General Hospital (ChiCTR1900023727). Informed consent was obtained prior to the start of the examinations.

### 2.2 UmNGS and analysis

The mNGS detection process includes an experimental operation and a bioinformatic analysis. The experimental procedure includes sample pretreatment, nucleic acid extraction, library preparation, and sequencing. Data quality control of bioinformatics analysis, human sequence removal, and alignment and identification of microbial species. First, we collected BALF (0.5 ml) and added 1 g of glass beads (0.5 mm) to a 1.5 ml tube. We performed the DNA extraction according to the manufacturer’s instructions for the TIANAMP Micro DNA Kit from TIANGEN BIOTECH (Beijing, China). The extracted nucleic acids were fragmented using the NextEra XT DNA Library Prep Kit (Illumina, San Diego, CA, United States) (Gu et al., 2019),followed by an end modification and primer addition. Finally, a library that met the sequencing requirements was obtained using PCR amplification. High-throughput sequencing was performed on the Illumina NextSeq sequencing platform. After obtaining the DNA sequence of the sample, quality control was performed, including adaper-trimming, low-complexity read filtering, and the removal of substandard reads. The remaining reads were compared to GRCh38, which was the human genome reference sequence, and the human-related reads were removed using Bowtie2 (BWA). The data were then classified into bacteria, fungi, viruses, and parasites using microbial reference genomes (ftp://ftp.ncbi.nlm.nih.gov/genomes/). From the identified microorganisms, colonization bacteria and sample-contaminating bacteria were excluded based on the baseline detection and background microorganism database (Chiu and Miller, 2019).

#### 2.2.1 UmNGS reporting criteria

The standard of interpretation of pathogen pathogenicity for the UmNGS report is: the threshold is generally at or above 8 RPM (reads per million), and for intracellular bacteria, a threshold of ≥1 RPM was considered meaningful.

### 2.3 QmNGS and analysis

#### 2.3.1 IC spiked into all specimens

Double-stranded DNA was synthesized using PCR and shared no significant homology to genomes of any known organisms, serving as a spiked-in IC (spike below instead), according to previous nucleotide sequences (Zhang et al., 2022; Zhou et al., 2022). The spikes were amplified and then mixed with magnetic beads for purification (Matridx, Cat# MD012). The amplification concentration was measured using a Qubit 4 fluorometer (Thermo Fisher Scientific). For the extraction of nucleic acids, spikes (0.02 ng/μl) were included in each sample.

#### 2.3.2 PCR-free library preparation and sequencing

400 µl of BALF was pipetted into a cartridge. The QmNGS wet lab workflow, including nucleic acid extraction, PCR-free library preparation, and purification, was performed automatically using the NGS master. The libraries were quantified using real-time PCR (chain) and pooled. Shotgun sequencing was performed using Illumina NextSeq. During each sequencing run, a negative control (NC, from healthy individuals, including human genomic DNA) and a positive control (a mixture of pathogen particles) were included for quality control, followed by trimming unnecessary adapter sequences and low-quality bases in the pipeline. Like UmNGS, sequences of human origin were identified using BWA (human reference genome) (Zhang et al., 2022). The host index was obtained by quantifying the number of human sequences on the molecular scale. If the host index was greater than 80% of similar samples, the human sequences were retained; otherwise, they were abandoned. The processed reads were mapped to NCBI GenBank to identify the microbial species, and the background microorganisms and contaminating bacteria were identified simultaneously.

#### 2.3.3 QmNGS reporting criteria

An identified microbial species was considered pathogenic when: (i) NC in the same sequencing run did not find the species or RPM (sample) was five times or more than RPM (NC); and (ii) for intracellular bacteria, RPM ≥ 1 was considered pathogen (Zhang et al., 2022; Zhou et al., 2022).

### 2.4 Data analyses

The final clinical diagnoses in this study were obtained by three experienced clinicians through a comprehensive evaluation of the clinical manifestations, traditional pathogen tests, CT imaging, BALF culture, and mNGS results of the patients. Microbial species detected by mNGS were considered pathogens only if they were consistent with the final clinical diagnosis.

### 2.5 Statistical analyses

A t-test, Wilcoxon signed rank test, or chi-square test was used to compare differences between UmNGS and QmNGS. All statistical results were analyzed using SPSS 22.0 software, and results with *P*-values < 0.05 were defined as statistically significant.

## 3 Results

### 3.1 Patient characteristics

**Figure 1:**
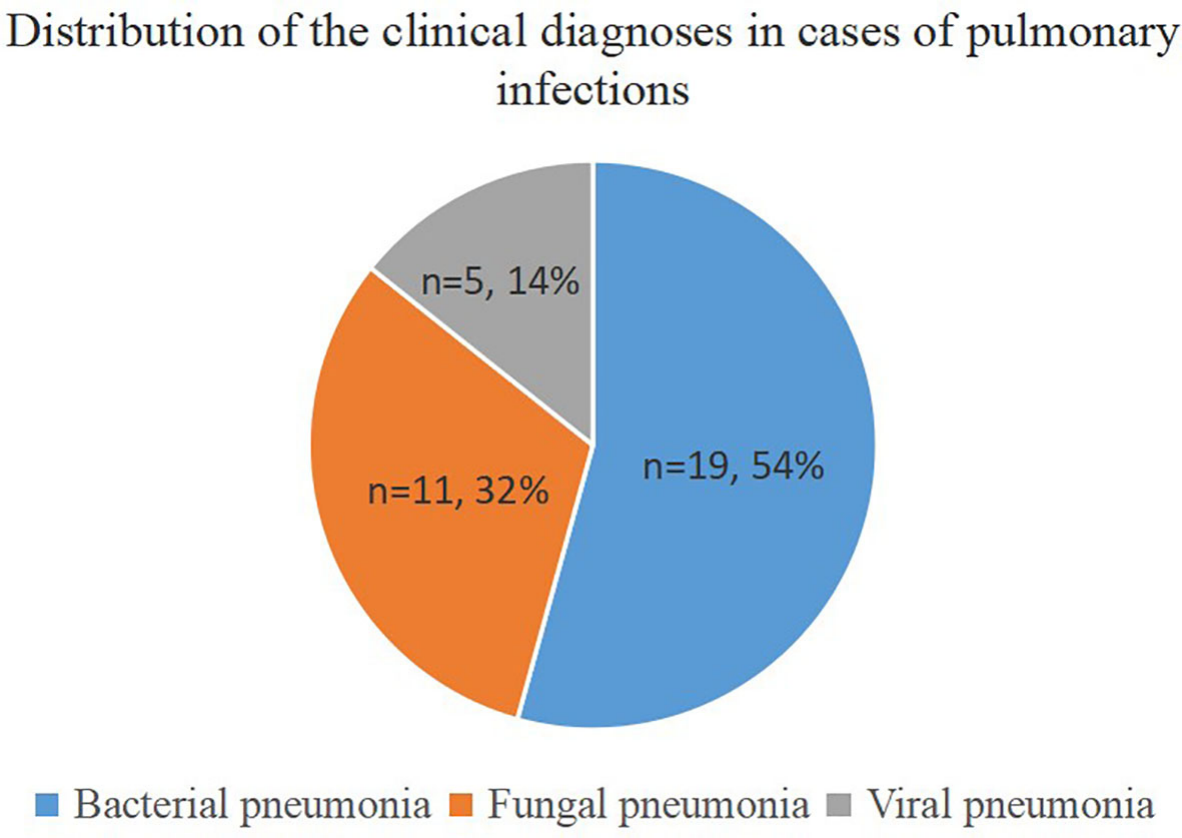
Distribution of clinical diagnoses in cases of pulmonary infections. The pie chart demonstrates the distribution of clinical diagnoses in 25 cases of pulmonary infection.

This prospective study included thirty-six patients, including nineteen men (52.8%) and seventeen women (47.2%), with an average age of 45 years. We found that 94.4% (n = 34) of the patients were immunocompromised, while only 5.6% (n = 2) had normal immune function. The immunocompromised patients suffered from hematological disorders or malignant tumors, and 67.6% (n=23) of them were diagnosed with a pulmonary infection. The final clinical diagnosis depended on clinical manifestations, imaging, traditional etiology tests, UmNGS and QmNGS, which included twenty-five (25/36, 69.4%) patients with a pulmonary infection and eleven (11/36 = 30.6%) patients with a nonpulmonary infection. Among the twenty-five patients with pulmonary infection, nineteen had non-mixed infections, and the rest (n = 6) had mixed infections. In the non-mixed infection group, ten patients had a single bacterial infection, seven had a single fungal infection, and two had a single viral infection. In the co-infection group, 33.3% (n = 2) of the patients were co-infected with one type of bacteria and one type of virus, 33.3% (n = 2) of the patients were co-infected with one type of fungus and a single bacterium, 16.7% (n = 1) of the patients were co-infected with one type of fungus and two types of bacteria, and 16.7% (n = 1) of the patients were co-infected with one type of fungus, three types of bacteria, and one type of virus (**Table 1**). In this study, thirty-five pulmonary pathogen infections were diagnosed in twenty-five patients with pulmonary infection, of which nineteen were diagnosed with bacterial pneumonia and eleven and five with fungal and viral pneumonia, respectively (**Figure 1**). After pathogen diagnosis, 84% of patients with pulmonary infection achieved relief in clinical symptoms through rational anti-infection treatment.

**Table 1:** Characteristics of the enrolled cases.

Age	45 ± 14.3
Sex	
Male	19 (52.8%)
Female	17 (47.2%)
Immune function	
Normal	2 (5.6%)
Defective	34 (94.4%)
Nonpulmonary infection	11
Pulmonary infection	25
Non-mixed infection	19
Single bacterial infection	10
Single fungal infection	7
Single viral infection	2
Mixed infection	6
Bacterial with one viral co-infections	2
Fungal with one bacterial co-infection	2
Fungal with two bacterial co-infections	1
Fungal with three bacterial and one viral co-infections	1

### 3.2 Pathogen species in patients with pulmonary infection

**Figure 2:**
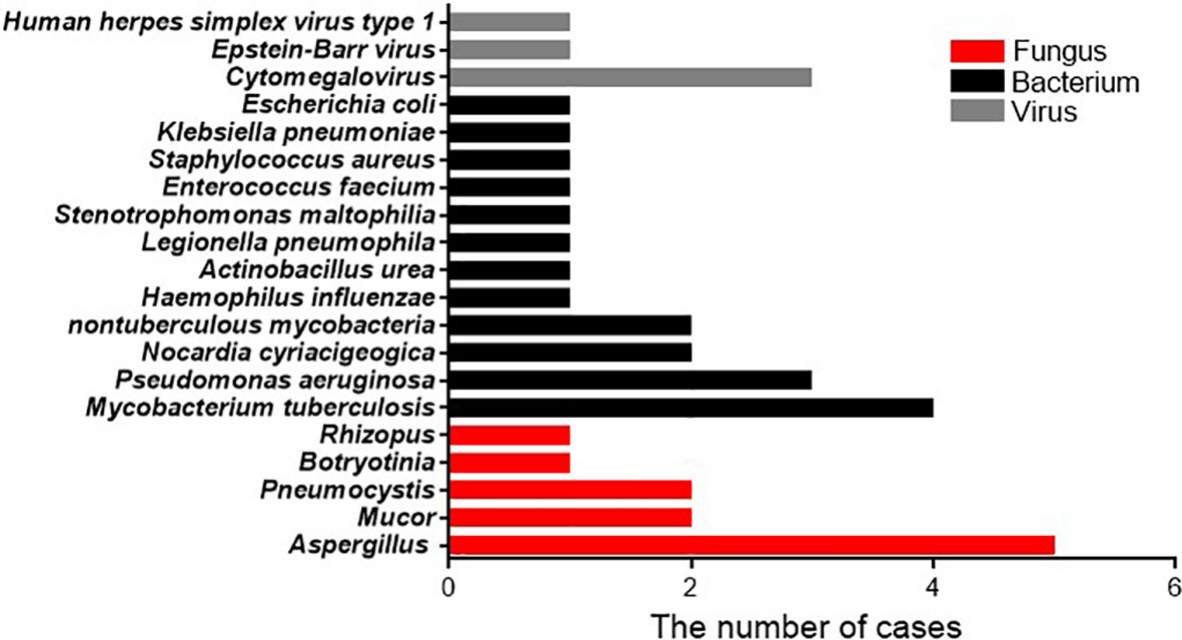
Pathogen occurrence in pathogen-infected patients. The X-axis shows the number of isolated pathogens in patients with clinically diagnosed pathogen-infected pneumonia.

In patients with bacterial infections diagnosed by mNGSs and conventional laboratory-based diagnostic testing, twelve species of bacteria were identified from nineteen isolated strains. The bacteria most commonly observed were *Mycobacterium tuberculosis*, which accounted for 4/19 infections (21.1%), followed by *Pseudomonas aeruginosa* accounted for 3/19 infection (15.8%), *Nocardia cyriacigeogica*, and *Nontuberculous mycobacteria*, all of which were observed in two cases (10.5%). The less common strains identified mostly belonged to gram-negative bacteria, including *Haemophilus influenzae*, *Actinobacillus urea*, *Legionella pneumophila*, *Stenotrophomonas maltophilia*, *Klebsiella pneumoniae*, *and Escherichia coli*, and only *Enterococcus faecium* and *Staphylococcus aureus* are gram-positive bacteria. Among the eleven patients with fungal infections, five species of fungi were detected based on mNGS and conventional diagnostic methods. The fungus most frequently detected was *Aspergillus* (n = 5, 45.5%), similar to a previous study (Yang et al., 2021). *Pneumocystis* and *Mucor* appeared with the same frequency (2, 18.2%). The relatively few isolated fungal species were *Botryotinia* and *Rhizopus* spp. For the five patients with viral infections, the strain most identified was *cytomegalovirus* (3, 60%), followed by *Epstein-Barr virus* and *human herpes simplex virus type 1* (**Figure 2**).

### 3.3 Detection performances of UmNGS and QmNGS in bronchoscopy and bronchoalveolar lavage fluid (BALF)

The comparison of UmNGS and QmNGS in the thirty-six patients with a suspected pulmonary infection is presented in **Table 2**. The sensitivity of UmNGS for diagnosing pulmonary infection was 76% (95% CI:54–90%), and the use of QmNGS was 72% (95% CI:50–87%). We did not find a significant difference in sensitivity between the two mNGS methods (P > 0.05). QmNGS was more specific (81.8%) than UmNGS (45.5%), with a difference of 36.3% (*P* = 0.219). Furthermore, we compare the difference in the LR for the two mNGS methods. The +LR values of QmNGS and UmNGS were 3.956 and 1.394, respectively, and the -LR values of QmNGS and UmNGS were 0.342 and 0.527, respectively. We did not find any difference in the LR between the two methods (*P* > 0.05).

**Table 2:** The detection performance of QmNGS and UmNGS in pulmonary infections.

	Sensitivity % (95% CI)	Specificity % (95% CI^a^)	+LR^b^	-LR^c^
QmNGS	72 (0.50-0.87)	81.8 (0.48-0.97)	3.956	0.342
UmNGS	76 (0.54-0.90)	45.5 (0.18-0.75)	1.394	0.527

a CI, confidence interval;

b +LR, positive likelihood ratio;

c -LR, negative likelihood ratio.

The above study identified nineteen cases of bacterial infection, eleven cases of fungal infection, and five cases of viral infection in patients with pulmonary infection. We compared the detection rates of QmNGS and UmNGS for the different pathogen species (**Table 3**). For bacterial infections, UmNGS and QmNGS detected seventeen cases (17/19, 89.5%) and sixteen cases (16/19, 84.2%), respectively. In eleven cases of fungal infection, the detection rates of the two methods were the same (7/11, 63.6%). In this study, five cases of viral infection were detected, all detected by QmNGS, while the results for UmNGS were positive in four cases (4/5, 80%).

**Table 3:** Comparison of the detection of pulmonary pathogens using the two different mNGS methods.

	Bacterial pneumonia	Fungal pneumonia	Viral pneumonia
QmNGS	16 (84.2%)	7 (63.6%)	5 (100%)
UmNGS	17 (89.5%)	7 (63.6%)	4 (80%)

### 3.4 Comparison of UmNGS and QmNGS analysis for co-detected pathogens

**Figure 3:**
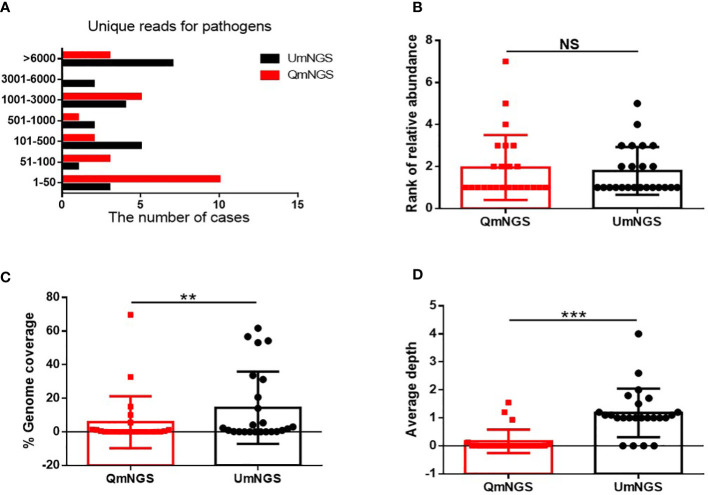
Comparison of UmNGS and QmNGS analysis for co-detected pathogens. The differences in the unique reads **(A)**, relative abundance ranking **(B)**, coverage **(C)**, and sequencing depth **(D)** of UmNGS and QmNGS for co-detected pathogens were compared, respectively. NS, no significant; **P < 0.05; ***P < 0.001.

As shown in **Figure 3** and **Supplementary Table 1**, 68.6% (n = 24) of the pathogens were detected using both detection methods. This study further explored whether there were differences in unique reads, relative abundance ranking, coverage, and sequencing depth of pathogens detected simultaneously using UmNGS and QmNGS. The number of unique reads of pathogens using UmNGS ranged from two to 104,564, and most were mapped to > 6000 (7/24, 29%). For QmNGS, the unique reads ranged from one to 149107, and 42% of the co-detected pathogens had fewer than fifty reads (10/24), while UmNGS accounted for 13% (**Figure 3A**). In other words, the number of unique reads of QmNGS pathogens was smaller than that of UmNGS; however, no significant differences were observed in the unique reads between QmNGS and UmNGS (*P* > 0.05). To further compare the differences between the two mNGS methods in unique readings, we performed a sample-to-sample comparison based on the data shown **Supplementary Table 1**. After statistical analysis, there was still no difference with *P* > 0.05. At the same time, we compared the differences in the relative abundance ranking of the co-detected pathogens between the two mNGS methods. The results also did not show significant differences (*P* = 0.638 > 0.05) (**Figure 3B**). Similarly, the difference of the relative abundance rankings between the two mNGS methods for each sample was compared, and no significant difference was found after the paired samples test (*P* > 0.05). Further comparisons of the genome coverage of 24 co-detected pathogens showed that the genome coverage of UmNGS was significantly higher than that of QmNGS (*P* < 0.05) (**Figure 3C**). In addition, a comparison of the differences in the average sequencing depth of the two methods for the same pathogen indicated that the average sequencing depth of the detected pathogen by QmNGS was significantly shallower than that by UmNGS, and the difference was statistically significant (*P* < 0.001) (**Figure 3D**).

### 3.5 Concordance between UmNGS and QmNGS assay in patients with pulmonary infection

**Figure 4:**
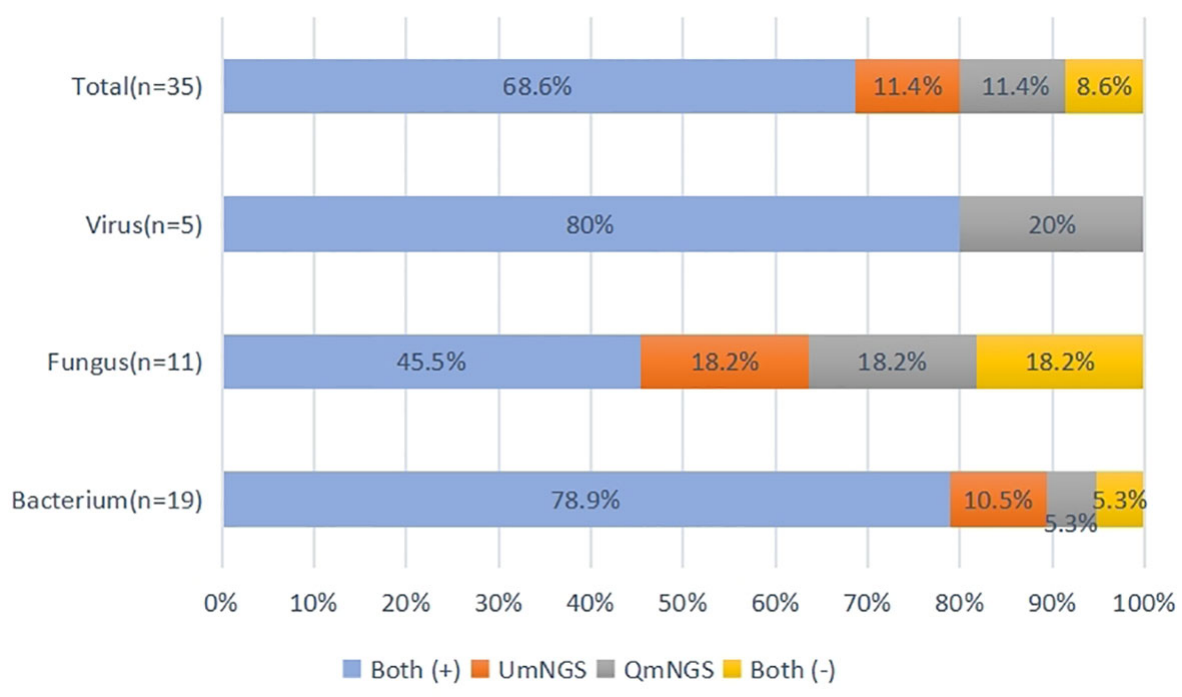
Concordance of QmNGS and UmNGS in the detection of a pathogen isolated from patients with a pulmonary infection. The coincidence rate of the two mNGS methods was 77.2%. Both (+), both methods detected the same pathogens in the same cases; both (-), both methods did not detect the same pathogens in the same cases.

**Supplementary Table 2** showed the thirty-five pathogen strains isolated from twenty-five patients with pulmonary infections and the detection performances of UmNGS and QmNGS for the strains level. We compared the consistency of UmNGS and QmNGS in the detection of these pathogens. The UmNGS and QmNGS assays were positive for 24/35 strains (68.6%) and negative for 3/35 strains (8.6%), while four strains (11.4%) were positive with QmNGS only, and four strains (11.4%) were positive with UmNGS only. The overall concordance rate between the two mNGS methods for pathogens detected in patients with pulmonary infection was 77.2%. To further describe the performance of the UmNGS and QmNGS concordance rate in different species of pathogens, we calculated the concordance rates of the two methods against bacteria, fungi, and viruses. The results showed that the concordance rates of different species of pathogens were different. The highest was for bacteria, up to 84.2%, followed by viruses, which was 80%, while the lowest concordance rate was for fungi at 63.7% (**Figure 4**).

### 3.6 The diagnostic performance of UmNGS and QmNGS in nonpulmonary infections

**Figure 5:**
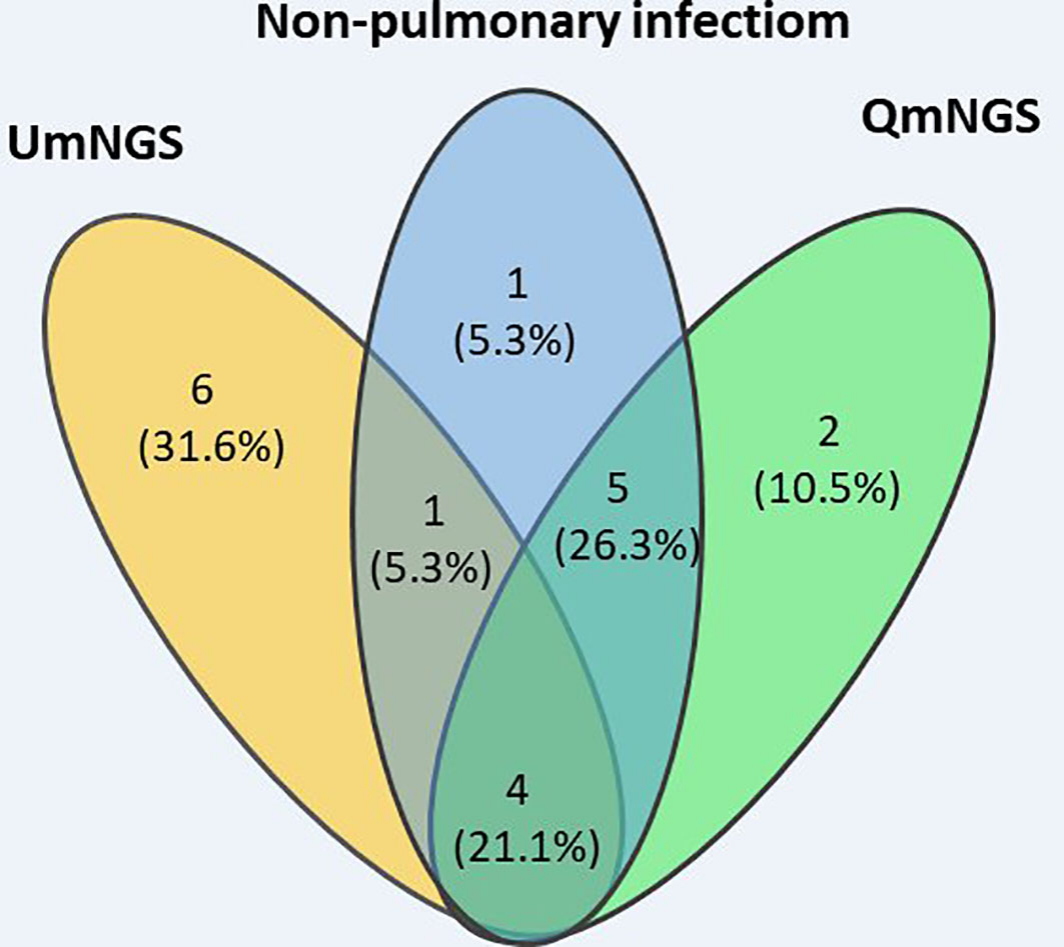
Wayne diagram describing the performances of QmNGS and UmNGS in patients with a nonpulmonary infection for pathogen detection. Blue represents eleven patients with nonpulmonary infection; yellow represents QmNGS; green represents UmNGS.

In this study, eleven cases of nonpulmonary infections were diagnosed based on relevant examinations. For nonpulmonary infections, the detection error rates for UmNGS and QmNGS were 54.5% (6/11) and 18.2% (2/11), respectively. However, the difference was not statistically significant (*P* = 0.219). Of the eleven patients with nonpulmonary infection, four cases were detected using QmNGS and UmNGS, and one case was incorrectly detected, as shown in **Table 4** and **Figure 5**. Nonspecific pathogens detected by the two mNGSs included bacteria, fungi, and viruses. The highest proportion was bacteria, which accounted for five out of ten, all detected using UmNGS, followed by viruses (4/10), using both UmNGS and QmNGS, while the least detected species was fungus (1/10) using only UmNGS (**Table 4**). Overall, the species of nonspecific pathogens detected using QmNGS were relatively uniform, while that using UmNGS was relatively diverse, in patients with a nonpulmonary infection. Furthermore, the diagnostic accuracy rate of QmNGS was higher than that of UmNGS, but the difference was not statistically significant.

**Table 4:** The diagnostic performance of UmNGS and QmNGS in nonpulmonary infections.

Sample No.	QmNGS	UmNGS
S5	Negative	*Staphylococcus hemolyticus*
S6	Cytomegalovirus	Cytomegalovirus Acinetobacter Pitt
S7	Negative	Negative
S9	Human polyomavirus 4	Negative
S15	Negative	*Staphylococcus hominis*
S16	Negative	Negative
S17	Negative	Negative
S23	Negative	Human respirovirus 3 *Staphylococcus hominis*
S25	Negative	Cryptococcus neoformans
S29	Negative	Negative
S36	Negative	*Streptococcus pneumoniae*

## 4 Discussion

Although mNGS has become a promising method for the diagnosis of infectious diseases, the limitations of mNGS testing, along with an increase in its clinical applications, have been highlighted. Institutions continue to optimize sequencing processes, database construction, and bioinformatics analyses to make mNGS more accurate and universal. Several optimized mNGSs are currently in the market. However, research on their performance in the clinical detection of pathogens is rare.

In this prospective study, we investigated the different performances of the two different mNGS methods with almost same costs, UmNGS and QmNGS, in the detection of infectious pathogens in BALF samples. We found that 54% of patients with pulmonary infection had bacterial infections, of which the most isolated strain was *Mycobacterium tuberculosis* (21.1%), which was inconsistent with common pathogens in hospital-acquired pneumonia (American Thoracic Society and Infectious Diseases Society of America, 2005). This may have been due to the different types of enrolled patients or the relatively small number of enrolled patients. Almost all enrolled patients (94.4%) were immunocompromised; they were much more susceptible to infection, and the pathogens may be uncommon and different from those of immunocompetent individuals (Thrikawala and Rosowski, 2020). Both QmNGS (72%) and UmNGS (76%) showed high sensitivity, similar to the results of a previous study (Huang et al., 2020). Our prospective study revealed that the specificities of UmNGS and QmNGS were 45.5% and 81.8%, respectively, and that the specificity of QmNGS was higher than that of UmNGS; however, the difference was not statistically significant. This may have been due to the limited number of patients that were enrolled in this study.

A key technical difference between UmNGS and QmNGS is the library preparation method. QmNGS uses a library preparation method that is reaction-free of PCRs. Compared to traditional library preparation dependent on PCR, the pathogen detection response time can be reduced to approximately 12 h, which is important for the rapid diagnosis and precise treatment of patients with life-threatening diseases. Furthermore, a study showed that the PCR-free library preparation method can reduce manual operation by technicians, thus reducing possible sources of contamination (Luan et al., 2021). Furthermore, QmNGS using NGSmaster, which automates the wet lab workflow, can further reduce exogenous contamination. The above two differences may explain why QmNGS has a higher specificity and lower diagnostic error rate in noninfectious pneumonia than UmNGS. A study suggested that the sequencing output of mNGS subsamples in the original DNA content of a library, and any bias introduced by the PCR library building process, would inevitably change the original information, which can then affect the relative abundance of pathogens in the sample (Gu et al., 2019). However, according to our results, a *p* value was greater than 0.05 after comparing the relative abundance rankings of the detected pathogens between the two mNGS methods. This may have been due to the relatively limited number of cases enrolled in our study or an improvement in the current PCR-dependent library preparation technology.

An inherent drawback of mNGS is that the predominant portion of microbial nucleic acids in most patient samples is the human host, accounting for > 95% of reads, which limits mNGS sensitivity (Yang et al., 2011). This shortcoming is unbiased and inherent in mNGS, and although this may be partially mitigated by host depletion (Hasan et al., 2016), it may lead to nonspecific clearance of pathogens, an amplification of the background microbiome, and interference with the interpretation of the results (Charalampous et al., 2019). Although this study revealed no differences were found between the two mNGSs regarding pathogen detection sensitivity, the specificity and negative LR tended to increase. Therefore, selective dehumanization of QmNGS did not cause a decrease in sensitivity compared with indifferent dehumanization of UmNGS but had a higher specificity. A study showed that the reads of the microorganisms identified by mNGS were not related to how the library was prepared but related to the concentration of host cells in the sample, which can decrease as the concentration of host cells increases (Luan et al., 2021). This was consistent with our finding that the pathogen reads were not significantly different between QmNGS and UmNGS.

Theoretically, our mNGS results are more reliable when the genome coverage and sequencing depth are higher (Li et al., 2021). Another study showed that samples from patients that were centrifuged to remove the cell precipitates before the library construction may reduce the proportion of the host genome proportion and increase the sequence coverage of pathogens significantly (Ji et al., 2020). Similarly, in our study, the sequencing depth and coverage of QmNGS were lower than those of UmNGS for co-detecting pathogens. However, neither the relative abundance ranking nor the sensitivity for detected pathogens were affected. However, the specificity showed an increasing trend, which may have been caused by the combined effect of the library construction method and the processing of human genes.

For the twenty-five patients with pulmonary infection, the concordance rate between the two mNGSs was 77.2% for pathogenic detection. Of the eleven patients with nonpulmonary infection, only four cases were detected using QmNGS and UmNGS. QmNGS detected viruses only, while UmNGS detected bacteria, viruses, and fungi, including *Staphylococcus* and *Cryptococcus neoformans*, which are classified more often as pathogens than viruses in clinics (Peng and Yang, 2021; Zhu et al., 2021). This may have provided a misleading anti-infection direction. Therefore, mNGS can detect not only the nucleic acids of the pathogens but also the nucleic acids of the colonized and contaminating pathogens, which is challenging to interpret. As the sensitivity of mNGS is improving, its specificity should be as high as possible to reduce any errors in diagnoses of nonpulmonary infections and has great significance for the rational use of antibiotics and avoidance of abuse. As shown in previous studies (Wang et al., 2019; Huang et al., 2020), mNGS has a higher false positive rate and higher cost compared to traditional pathogen detection methods, and traditional detection methods cannot be replaced.

As a limitation, due to the insufficient number of subjects included in this study, a true statistical difference between the two mNGS assays could not be established. However, the application value of QmNGS is promising.

In summary, this study conducted a comprehensive comparison of QmNGS and UmNGS in the detection of pathogens in BALF to understand the differences between the different mNGS technologies and offer a basis for the clinical selection of the diverse types of mNGS.

## Data availability statement

The data presented in the study are deposited in the NCBI, accession number PRJNA940347 and PRJNA936669.

## Ethics statement

The studies involving human participants were reviewed and approved by Ethics Review committee of Tianjin Medical University General Hospital (ChiCTR1900023727). The patients/participants provided their written informed consent to participate in this study.

## Author contributions

YW and JF contributed to the research design and revision of the manuscript. ZZ and XC drafted the research protocol, analyzed the results, drafted the manuscript and deposited data in an acceptable repository. All authors approved the submitted version and agreed to be responsible for all aspects.
